# Diverse activities and biochemical properties of amylase and proteases from six freshwater fish species

**DOI:** 10.1038/s41598-021-85258-7

**Published:** 2021-03-11

**Authors:** Chamaiporn Champasri, Suthathip Phetlum, Chanakan Pornchoo

**Affiliations:** Department of Biochemistry, Faculty of Science, Khon Kaen University, Khon Kaen, 40002 Thailand

**Keywords:** Biochemistry, Zoology

## Abstract

This study investigated the biochemical properties, enzyme activities, isoenzyme pattern, and molecular weight of three types of digestive enzyme from six freshwater fish species: *Puntius gonionotus* (common silver barb), *Puntioplites proctozysron* (Smith’s barb), *Oreochromis niloticus* (Nile tilapia), *Hemibagrus spilopterus* (yellow mystus), *Ompok bimaculatus* (butter catfish), and *Kryptopterus geminus* (sheatfish). The optimum pHs for amylase and alkaline protease activities were 7.0–8.0 and 8.0–10.0, and the optimum temperatures were 45–60 °C and 50–55 °C, respectively. A pepsin-like enzyme was detected in all three carnivorous fishes (*Ompok bimaculatus*, *Kryptopterus geminus*, and *Hemibagrus spilopterus*) with optimum reaction pH of 2.0 for each and optimum reaction temperatures 50–55 °C. In optimum reaction conditions, the amylase and alkaline protease from *Puntioplites proctozyron* showed the highest activities. Lower activities of all enzymes were observed at temperature (29 °C) of Lam Nam Choen swamp than at the optimum reaction temperatures. The fish species contained one to three and five to eight isoforms of amylase and alkaline protease, respectively, with molecular weights from 19.5 to 175 kDa. Both the alkaline proteases and amylases were stable in wide pH and temperature ranges.

## Introduction

Amylase and protease are important enzymes in cellular metabolism in plants, animals, and microorganisms. Both enzymes function to catalyze degradation of macromolecules into small building blocks, which are subsequently used to produce energy or to synthesize other biomolecules inside cells. Extracellular digestive enzymes present in the digestive tract of animals or in the digestive system of carnivorous or insectivorous plants^1,2^ also have a crucial role in digestion of macromolecules from food.

Proteases catalyze the breakdown of proteins into small peptides and amino acids. They are found in all living organisms. Based on the optimum pH for enzymatic activity, these enzymes are classified into two groups—acidic and alkaline proteases. The former enzymes, such as pepsin, function in acidic conditions with optimum pH around 2.0–4.0 and are mostly found in the stomach of humans and animals. Alkaline proteases are present in the small intestine of humans and the digestive system of animals. This group includes chymotrypsin and trypsin, which have optimum reaction pH values ranging from 8.0 to 12.5.

Amylases catalyze the hydrolysis of polysaccharides such as starch and glycogen into short-chain sugars. These enzymes are ubiquitous and play an important metabolic role. According to catalytic function and enzyme structure, amylases are classified into three types: α-amylase (EC 3.2.1.1), β-amylase (EC 3.2.1.2), and γ-amylase (EC 3.2.1.3). α-Amylase is a metalloenzyme requiring calcium ions for catalytic function. This enzyme randomly hydrolyzes α-1,4-glycosidic bonds, releasing maltose and glucose^3^. β-Amylase is a calcium-independent enzyme that catalyzes the hydrolysis of α-1,4-glycosidic linkages at the nonreducing end of starch molecules, yielding a maltose unit. γ-Amylase catalyzes the hydrolysis of the last α-1,4-glycosidic bond at the nonreducing end of starch yielding one glucose unit; γ-amylase is mostly active in acidic conditions.

Due to the rapid growth of microorganisms, which enables high-yield enzyme production, microbial enzymes are widely used in various industries such as the manufacture of detergent, paper and pulp, textiles, and food and animal feed^4^. Nevertheless, there are drawbacks of some bacterial enzymes, such as low stability at high temperature (except for thermophilic bacteria) or a narrow pH range for activity. Therefore, novel enzymes with high activity and high stability at broad ranges of pH and temperature are still required for a wide range of uses. Studies of protease and amylase have been reported in plants^5–7^, insects^8,9^, microorganisms^10,11^ and fish^12–18^. The data report various enzyme activities, biochemical properties, and molecular weights of amylase and protease. The optimum pH values for catalytic activity of acidic proteases, alkaline protease, and amylase are in the range 2.5–3.0, 7.5–12.5 and 7.0–9.0 respectively, and the optimum temperature varies between 25 and 70 °C. Molecular weights range from 18 to 101 kDa and 18 to 139.5 kDa for protease and amylase, respectively. The number of isoenzymes (the enzymes differ in amino acid sequences and molecular sizes but catalyze the same reaction) varies from one to eight, dependent on the species from which the enzymes are derived. Noticeably, almost all bacteria and plants contain only one isoform of amylase and protease, but fishes and shrimps contain many isoforms. In addition, different species have isoenzymes of different molecular weight^16,19^. However, almost all recently available data on fish digestive enzymes are from omnivorous fish. Little information has been reported about these enzymes in carnivorous or herbivorous fish.

Cyprinidae, Siluridae, Bagridae, and Cichlidae are fish families widely distributed in North America, Africa, and Southeast Asia^20^. *Puntius gonionotus* (common silver barb) and *Puntioplites proctozysron* (Smith’s barb) belong to the Cyprinidae; *Ompok bimaculatus* (butter catfish) and *Kryptopterus geminus* (sheatfish) belong to the Siluridae; *Hemibagrus spilopterus* (yellow mystus) belongs to the Bagridae; and *Oreochromis niloticus* (Nile tilapia) belongs to the Cichlidae. All six species are important for food, and economically. The types and number of fish species in a locality depend on various factors such as the abundance of the ecosystem and the local management of water resources. The growth rate of each fish species affects the abundance and fish diversity.

Little is known on the digestive systems of freshwater fishes, therefore this study aimed to investigate the biochemical properties of digestive enzymes—amylase and protease—from six freshwater fish species. The enzyme activities in optimum and environmental conditions were determined and compared. Moreover, the isoenzyme patterns and their molecular weights, as well as the enzyme stabilities, were analyzed. This study will help understanding of the digestive enzymes of each fish species, not only for sustainable fish management, but also for improvement of fish feed formula, in order to increase the growth rate of fish and reduce feed cost and cultivation time in aquaculture.

## Results

### Fish samples

Carnivorous fishes *Om. bimaculatus*, *K. geminus*, *H. spilopterus*; herbivorous fishes *Puntius gonionotus* and *Puntioplites proctozysron*; and omnivorous fish *Or. niloticus* were selected for this study. Details of body length, body weight, digestive tract weight, and calculated digestive somatic index (DSI) values are indicated in Table 1. A high DSI value indicates high digestive capability. *P. gonionotus*, *Or. niloticus*, and *P. proctozysron* exhibited high digestive enzyme activity; the two former species displayed a high DSI value, but the latter did not.

**Table 1:** Data of body length, body weight, digestive tract weight, and calculated digestive somatic index of fishes.

Fish species	Body length (cm)	Body weight (g)	Digestive tract weight (g)	DSI
*Ompok bimaculatus*	19.30 ± 1.8	55.02 ± 4.1	1.44 ± 0.3	2.73 ± 0.6
*Kryptopterus geminus*	14.58 ± 0.60	17.10 ± 1.75	0.43 ± 0.08	2.53 ± 0.53
*Hemibagrus spilopterus*	21.20 ± 1.29	73.86 ± 6.43	3.88 ± 1.24	3.44 ± 1.04
*Puntius gonionotus*	15.15 ± 1.6	48.84 ± 13.9	3.65 ± 1.1	7.06 ± 0.2
*Oreochromis niloticus*	23.08 ± 3.2	203.47 ± 62.6	11.13 ± 1.4	6.46 ± 1.8
*Puntioplites proctozysron*	13.08 ± 1.67	31.63 ± 14.69	0.97 ± 0.28	3.07 ± 1.33

Values are mean ± SEM of five to eight specimens of each fish species.

### Optimum pH and temperature for enzyme activity

**Figure 1:**
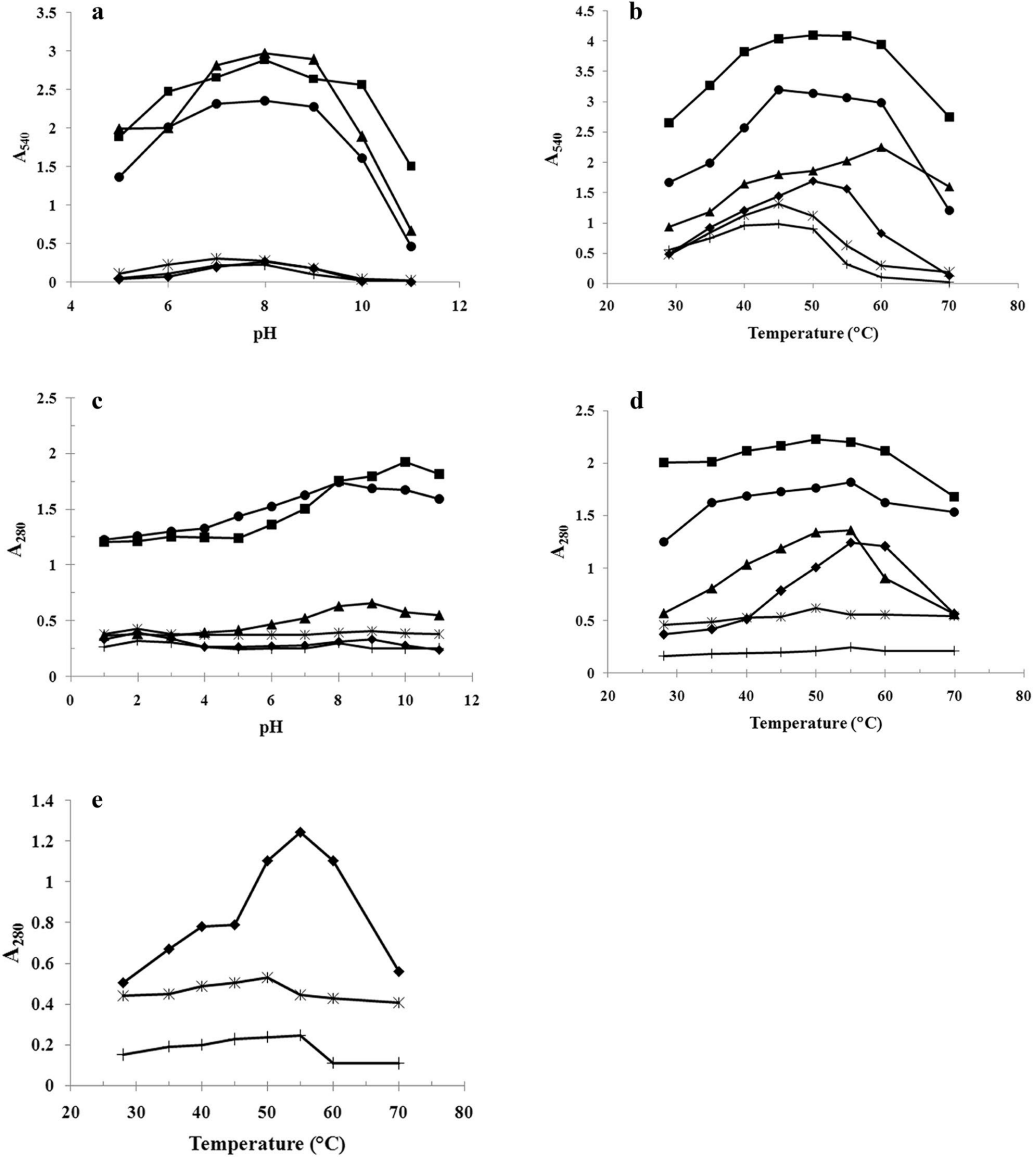
Optimum pH and optimum temperature for amylase (**a**, **b**), alkaline protease (**c**, **d**), and optimum temperature of pepsin-like enzyme (**e**) (-■-, *Puntius gonionotus*; -●-, *Puntioplites proctozysron*; -▲-, *Oreochromis niloticus*; -◆-, *Hemibagrus spilopterus*; -*-, *Kryptopterus geminus*, -**+ **-; *Ompok bimaculatus*).

Time course assays showed a linear relationship between enzyme activity and incubation time when using 20 or 25 μl of crude enzyme preparation containing mixtures of isoenzymes extracted from the digestive tract of each fish species for the detection of amylase or protease activity (data not shown). Therefore, an incubation time between 5 and 20 min was chosen for testing enzyme activity. The enzymes from the different fish species showed various optimum pHs and temperatures for enzymatic activity. Almost all of the amylases showed the same optimum pH for activity, pH 8.0, except for *K. geminus* amylase, for which the optimum pH was 7.0 (Fig. 1a). The maximum alkaline protease activity was observed at different pH values for the enzymes from different fishes (Fig. 1c).

The optimum temperatures for enzyme activity in this study were in the range 45–60 °C, 50–55 °C, and 50–55 °C for amylase, alkaline protease, and pepsin-like enzyme, respectively (Fig. 1b,d,e). No correlation between fish families and optimum temperature for enzyme activity was observed. Interestingly, we found thermostable enzymes in *P. gonionotus* and *P. proctozysron*. Both amylases and alkaline proteases from these fish showed high activities in a broad temperature range, particularly the alkaline proteases, whose activities were > 84% at 35 to 60 °C compared with the maximum activity at the optimum temperature (defined as 100% activity). This result suggests that these enzymes are potential candidates for use in broad applications.

### Comparison of enzyme activity

**Figure 2:**
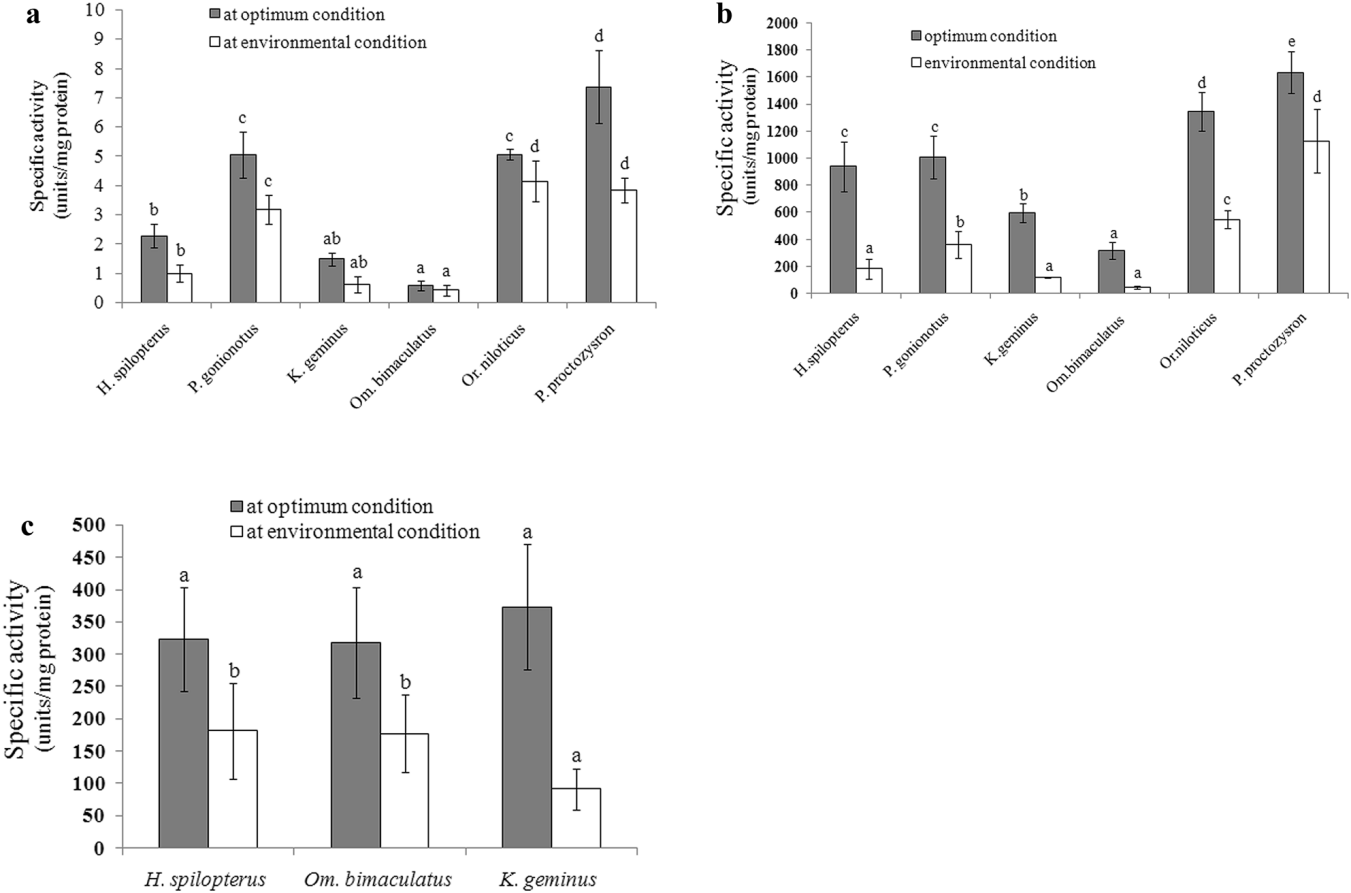
Activity comparisons of amylase (**a**), alkaline proteases (**b**), and pepsin-like enzyme (**c**) (Data are expressed as Mean ± SD (n = 3–8). The differences among means of the same condition (optimum condition or environmental condition) were compared by Duncan’s multiple range tests. Different small letters indicate the significant differences at *P* value < 0.05.

Study of enzyme activity at different temperatures helps us to understand food digestion capacity of fishes. Fish are cold-blooded—their temperature depends on the water temperature. However, determination of digestive enzyme activity at the water temperature of the swamp, pond, or aquarium has rarely been reported. In this study, digestive enzyme activities determined in optimum and environmental conditions were compared among six fish species. In optimum conditions, *Puntioplites proctozysron* showed the highest activities of both amylase and protease, with specific activities of 7 ± 1.2 units mg^−1^protein and 1634 ± 115.5 units mg^−1^protein, respectively; the next highest activities were shown (in order) by the enzymes from *Or. niloticus*, *Puntius gonionotus*, and *H. spilopterus*. Low activities of both enzymes were observed in *K. geminus* and *Om. bimaculatus* [specific activities less than 1.5 units mg^−1^protein (for amylase) and 595 units mg^−1^protein (for alkaline protease)] (Fig. 2). Pepsin-like enzymes were apparent in the carnivorous fishes *H. spilopterus*, *K. geminus* and *Om. Bimaculatus*, with specific activities < 375 units mg^−1^ protein. The digestive enzyme activities were also investigated at swamp temperature (29 °C), to evaluate food digestion capability. The enzyme activities were 42%–90%, 13%–69%, and 24%–56% for amylase, alkaline protease, and pepsin-like enzyme, respectively, compared with those determined in optimum conditions, suggesting that the proteases, particularly alkaline protease, were more sensitive to temperature than amylase. Moreover, we found that the carnivorous fishes displayed lower amylase and alkaline protease activities than the omnivorous and herbivorous fishes in both temperature conditions.

### Isoenzyme patterns and molecular weight

Amylases, alkaline proteases, and pepsin-like enzyme were separated by SDS-PAGE and the molecular weights of these enzymes were analyzed. Using 12% SDS gels revealed different patterns of the three enzymes in different fish species. In this zymographic analysis, clear bands on the dark background of the stained gel indicate the position of an enzyme that can hydrolyze substrate.

**Figure 3:**
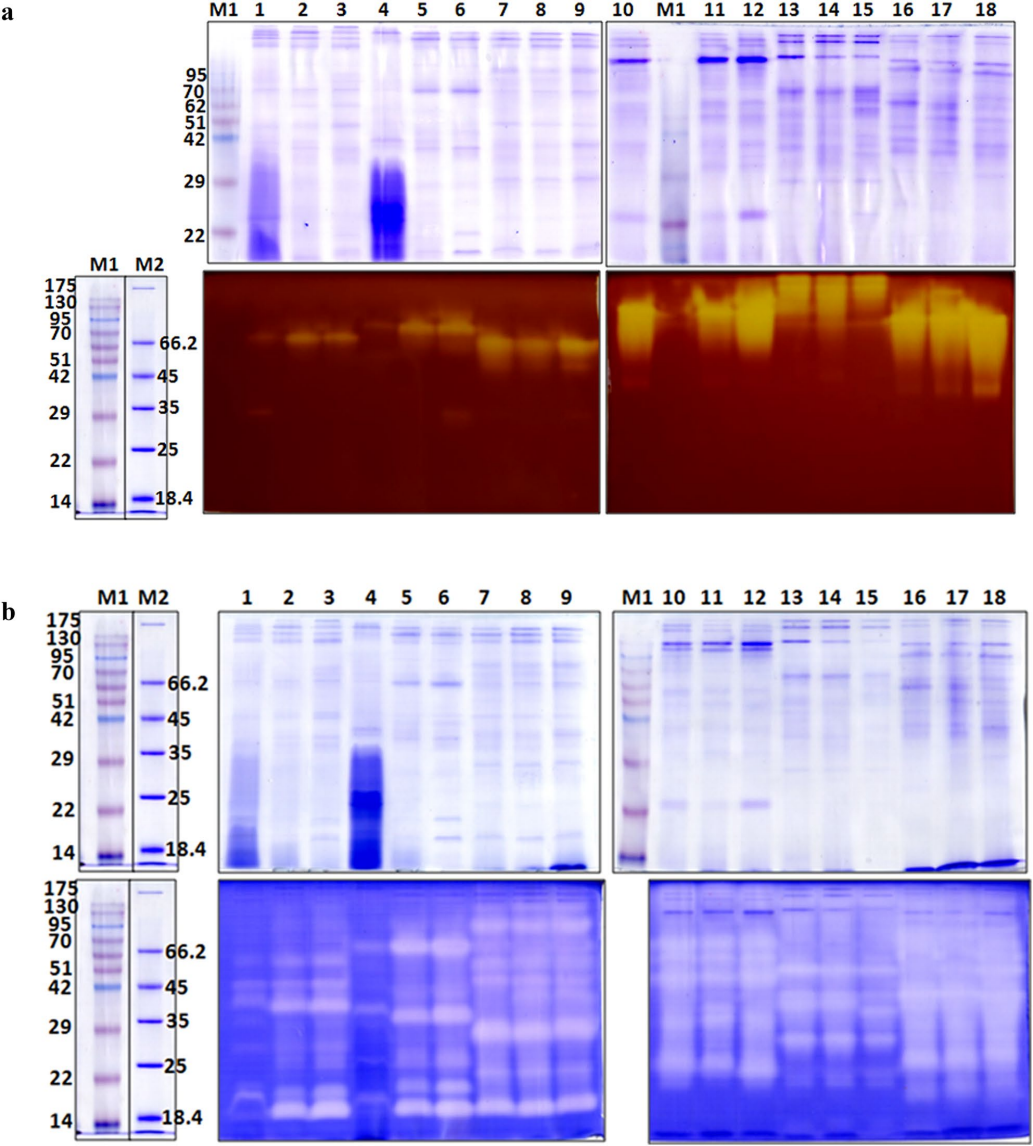
SDS gels stained with Coomassie brilliant blue R-250 and zymograms of amylase (**a**), alkaline proteases (**b**) (Lane M1; Pink plus prestained protein ladder (GeneDireX), lane M2; unstained protein molecular weight marker (Thermo Scientific), lanes 1–3; *Ompok bimaculatus* (n6, n7, n8), lanes 4–6; *Kryptopterus geminus* (n6, n7, n8), lanes 7–9; *Hemibagrus spilopterus* (n1, n2, n4), lanes 10–12; *Puntius gonionotus* (n5, n7, n8), lanes 13–15; *Oreochromis niloticus* (n5, n7, n8), lanes 16–18; *Puntioplites proctozysron* (n1, n2, n3)).

The six fish species contained one to three isoforms of amylase with estimated molecular weights from 35 to > 175 kDa (Fig. 3a and Table 2). The number of isoforms of alkaline protease ranged from five to eight in the six fish species (Fig. 3b); the molecular weights were from 19.5 to 130 kDa, as indicated in Table 2. Zymographic results corresponded to enzyme activity analysis data; enzymes with high activity exhibited strong, clear bands, whereas enzymes with low activity showed weak bands on the gels. In the case of pepsin-like enzyme, no clear enzyme bands were observed with 100 μg of total protein for samples from *Om. bimaculatus* or *K. geminus* although they exhibited specific activities of 318 ± 85.9 and 373 ± 97.4 units mg^−1^protein, respectively (Fig. 2c). One clear band was detected for *H. spilopterus* pepsin-like enzyme, with molecular weight 24 kDa (data not shown). The failure to detect pepsin zymographically might be because of the low amounts of sample used, and/or because the enzyme did not refold properly when SDS was washed out of the gel.

**Table 2:** Number of isoenzyme and the estimated molecular weight of amylase, alkaline protease and pepsin-like enzyme.

Fish species	Amylase		Alkaline proteases		Pepsin	
	No	MW (kDa)	No	MW (kDa)	No	MW (kDa)
*Ompok bimaculatus*	1–2	70, 35	8	57, 45, 37, 35, 27, 25, 21, 19.5	ND	ND
*Kryptopterus geminus*	1–2	82.5, 35	6	66.5, 41, 35, 26, 21, 19.5	ND	ND
*Hemibagrus spilopterus*	1–2	66, 35	8	130, 95, 56, 45, 40, 29, 23, 19.5	1	24
*Puntius gonionotus*	1–2	95, 49	5	70, 62, 46.5, 34, 24	–	–
*Oreochromis niloticus*	3	> 175, 175, 152	6	51, 38.5, 37, 27.5, 23, 21	–	–
*Puntioplites proctozysron*	2–3	152, 82, 49	7	80, 67, 62, 51, 35.5, 25, 20	–	–

ND: not detectable.

### Enzyme stability

**Figure 4:**
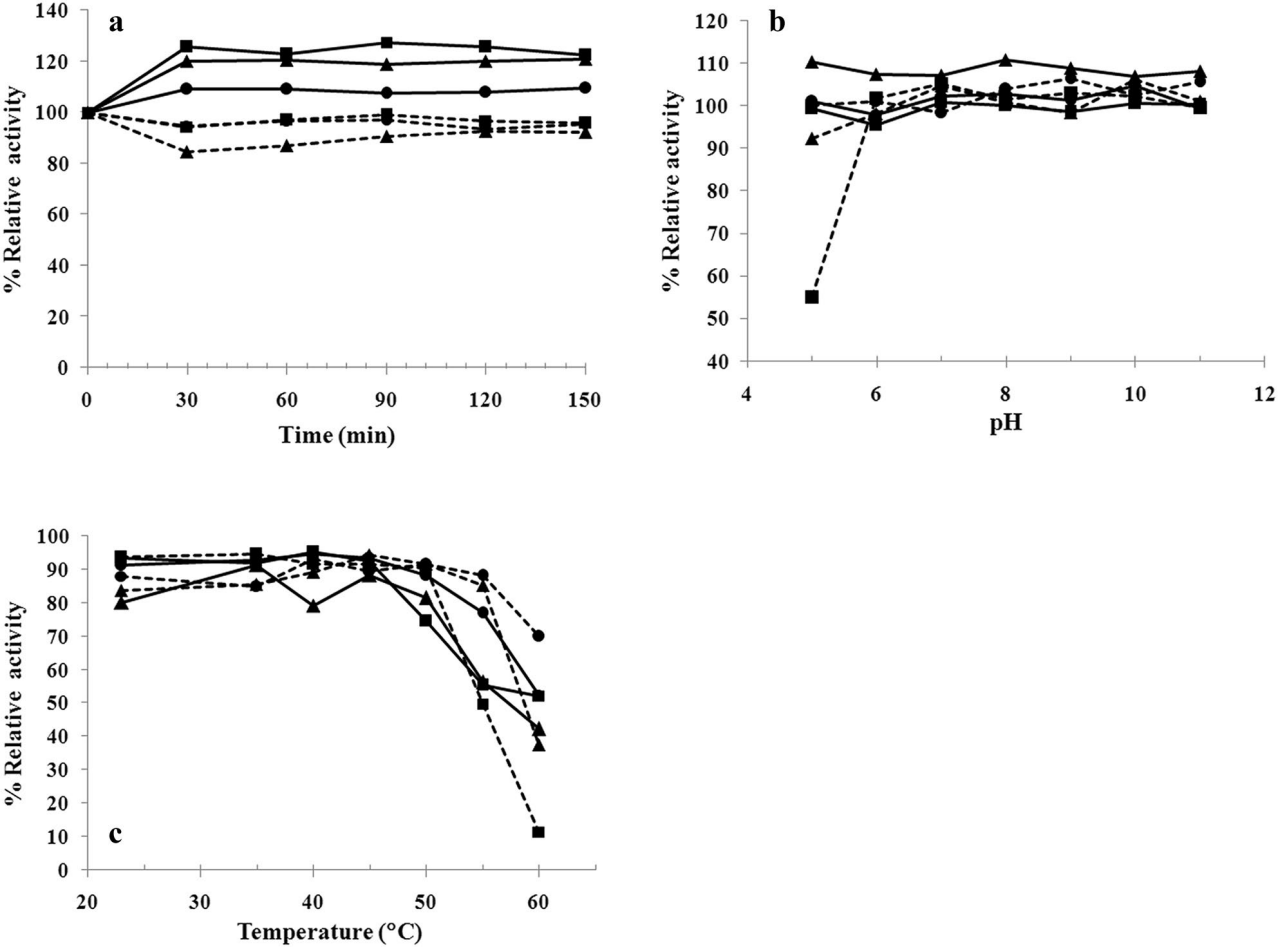
Effects of incubation time (**a**), pH (**b**) and temperature (**c**) on amylase and alkaline proteases activities (-■-, *Puntius gonionotus*; -●-, *Puntioplites proctozysron*; -▲-, *Oreochromis niloticus*. Dashed lines represent the relative activity of amylase, whereas solid lines represent the relative activity of alkaline proteases).

The effects of incubation time, pH, and temperature on enzyme activity were determined using crude enzymes samples to help understand more biochemical properties of the enzymes that will be useful for further study and application. Enzymes from three fish species (*P. gonionotus*, *P. proctozysron*, and *Or. niloticus*) exhibiting high activities of both amylase and alkaline protease in broad pH and temperature ranges were used for this experiment. Figure 4a shows the enzyme activities at different times of incubation in 0.2 M Tris-HCl buffer, pH 8.0. All three species exhibited the same pattern of enzyme stability. In the first 30 min of incubation, the activities of the alkaline proteases increased to 108%–125% and then maintained activities > 100% until 150 min; while amylase activities decreased to 94%–84% before a slight increase to stabilize at > 92%. Note that 100% relative activity was defined as the enzyme activity assayed at the same conditions, without a preincubation step; after preincubation, some of the enzymes showed higher activity.

Considering the effect of pH, most of the enzymes from the tested fish species revealed high stability in a broad pH range (from 5.0 to 11.0) with enzyme activity up to 110% (Fig. 4b). However, in the case of *P. gonionotus*, the relative amylase activity decreased and remained at 55% at pH 5.0. At pH 2–5, acid-induced denaturation of protein often occurs. Decreasing pH by adding acids (buffer pH 5.0 in this case) to the enzyme solution (in extraction buffer, pH 7.5) causes the side chain –COO^-^ converted to –COOH resulting in changes in ionic interaction between side chains, leading to unfolding and denaturation. The *P. gonionotus* amylase seems not able to refold after adding of an optimum buffer for activity assay. Figure 4c reveals the effect of temperature after incubation of enzyme for 1 h at 25–60 °C. Both the amylases and alkaline proteases from all three species exhibited high residual activities (> 80%) after incubation at 25 to 50 °C, but the activities dropped dramatically, to 11%–70%, after incubation at higher temperature, most notably for *P. gonionotus* amylase.

## Discussion

This research studied the biochemical properties of digestive enzymes from some freshwater fishes. The enzymes from the different fish species had various optimum pHs and temperatures for enzymatic activity. These data correlated with results from seven cyprinid fishes^16^ and *Or. niloticus*^21^. These results suggest that the enzymes may share conserved amino acid residues at the active site^22,23^. In other organisms such as bacteria (*Bacillus cereus*, *Bacillus amyloliquefaciens* BH072, and *Aeribacillus pallidus* C10), shrimp (*Cherax albidus*, *Penaeus californiensis* Holmes 1900, *Penaeus vannamei* Boone 1931, and *Penaeus stylirostris* Stimpson 1874), and other fish species (*Osteochilus hasselti*, *Labiobarbus spilopleura*, *Osteochilus lini*, *Cyclocheilichthys repasson*, *Cyclocheilichthys apogon*, *Puntius brevis, Symphysodon aequifasciata*, *Polyodon spathula, Limia vittata*, and *Gambusia punctata*), the amylases exhibit different optimum pH values, 6.0, 7.0, 8.0, or 9.0^10,21,24,25^, whereas the proteinases or alkaline protease reveal wide ranges of optimum pH from 7.0 to 12.5^13,19,21,24,26–28^. In the present study, different optimum pH values were observed for alkaline protease, with the values varying from 8.0 to 10.0 depending on the fish species. Moreover, acidic proteases were also observed. Low activity of this pepsin-like enzyme was observed in *H. spilopterus*, *Om. bimaculatus*, and *K. geminus*. However, the maximum activity of the enzyme was detected at pH 2.0.

Fish are cold-blooded; thus, the water temperature influences their body temperature, growth rate, and food consumption^29–31^. The growth rate is associated with digestive capability, which is derived from digestive enzyme activity. Study of the digestive enzyme activity at different temperatures will help with sustainable development of aquaculture. In this study, we found that all six species of freshwater fish showed low digestive enzyme activity at environmental temperature (29 °C), particularly for alkaline proteases whose activities dropped to 13%–69% of those in the optimum conditions. In addition, the carnivorous fishes displayed lower amylase and alkaline protease activities than the omnivorous and herbivorous fishes.

Isoenzyme analysis and molecular weight determination of amylase and protease enzymes have been performed in bacteria, shrimp, and some species of fishes. Noticeably, bacterial enzymes display only one isoform with molecular weight ranging from 28 to 68 kDa, whereas fish and shrimp contain many isoforms^10,12,13,16,19,21,24–28^. The several isoforms of digestive enzymes seen in digestive tracts of fish may derive from the floral bacteria^32^. However, more experiments have to further investigate bacterial enzymes such as the expression level, biochemical properties, and molecular weights compared to enzyme extracts from digestive tracts of fish.

In the present study, the number of isoenzymes of proteolytic and amylolytic enzymes ranged from one to eight and two to eight with molecular weights between 12 and 160 kDa and 26 and 175 kDa, respectively. In the case of pepsin-like enzymes, although three fish species in this study exhibited specific activities around 320–375 units mg^−1^protein, only one such enzyme (that from *H. spilopterus*) could be detected on a zymographic gel, and the band was very faint.

pH and temperature stabilities have been reported for bacterial α-amylases^10,11,33,34^ and bacterial proteases^33,35^. Amylases from *Bacillus* showed high stability at pHs between 5.0 and 10.0, and temperatures between 20 and 100 °C with residual activity > 70%; proteases had residual activity > 70% at pH 8.0 to 12.0 and 30 to 60 °C. For marine fish, the digestive enzymes from Atlantic salmon (*Salmo salar*)^36^ and thornback ray (*Raja clavata*)^37^ have been reported. For Atlantic salmon, trypsin showed high stability with > 92% residual activity after incubation at 37 °C for 24 h, and alkaline protease from thornback ray was highly stable at pH 5.0 to 11.0 and 4 °C. However, little information has been reported on stability of digestive enzymes from freshwater fish. Seven species of fishes including of *P. gonionotus* have been reported the biochemical properties of digestive enzyme^16^. However, molecular weight and enzyme stabilities have not been determined. Chaijareon and Thongruang (2016) determined the stability of amylase and protease from *Or. niloticus*. They found that the protease exhibited 50% residual activity after 360 min of incubation at 60 °C at pH 8.0, while the amylase activity increased fivefold in the first 30 min and declined three-fold of its initial activity after 90 min. In contrast, our experiments showed that both amylase and alkaline protease from *Or. niloticus* and also those from *P. proctozysron* and *P. gonionotus* exhibited high stability at a wide range of pH (6.0–12.0) and temperature (25–50 °C), retaining more than 95% and 75% of their initial activities, respectively. Moreover, the enzymes were stable on incubation in buffer pH 8.0 at room temperature (23 °C) for 150 min. High stability of amylase and alkaline protease, digestive enzymes from freshwater fishes, was found in this study and is good evidence for further study and application.

## Materials and methods

### Materials

Casein (sodium salt, from bovine milk, cat.no. C-8654) and starch (from rice, cat.no. S-7260) were purchased from Sigma-Aldrich, USA. Quick Start 1 × Bradford dye reagent (cat.no. 5000205) was from Bio-Rad, Bio-Rad laboratories, Hercules, California. Acrylamide (cat.no. 17-1302-02) and N, N′-methylenebisacrylamide (cat.no. 17-1304-02) were purchased from Amersham Biosciences, USA. Sodium dodecylsulfate (cat.no. DB0485), soluble starch (cat.no.SB0904) and β-mercaptoethanol (cat.no. MB0338) were from Bio Basic Canada Inc. 3,5-Dinitrosalicylic acid was purchased from Fluka, USA. Chemical reagents for preparation of buffers were purchased from Carlo Erba (France), Scharlau (Barcelona, Spain), and Ajax Finechem (MA, USA).

### Sample collection and preparation

Samples were collected as part of a fish faunal survey in Lam Nam Choen swamp, Nongruea district, Khon Kaen province, Thailand, by using nets. Fish were killed in ice without using any chemical agent. The condition did not severely distress or cause lasting harm to sentient fish. The body weight and body length were recorded. Whole digestive tracts were dissected and weighed before calculation of DSI [(digestive tract weight/body weight) × 100]. The digestive tracts were subsequently rinsed with distilled water and then homogenized on ice by uses of pestle and mortar with a buffer (0.05 M Tris-HCl, pH 7.5) using 1 g of digestive tract per 3 ml of buffer. The homogenized samples were centrifuged by uses of Rotina 380R centrifuge (Hettich) at 22,000 × *g* at 4 °C for 30 min. Supernatants containing digestive enzymes were subjected to further study or kept at − 20 °C until use. Determination of protein content was performed by using Bradford reagent^38^. Bovine serum albumin was used for the calibration curve of protein concentration.

All methods were carried out in accordance with relevant guidelines and regulations. All experimental protocols and the care and use of experimental animals complied with animal welfare laws of Thailand, and guidelines and policies approved by ThaiIACUC (permit reference number U1-04,584-2559).

### Time course assay

To determine the initial velocity of enzymatic reaction, the enzyme activity was observed after incubation of enzyme with substrate for 5 to 30 min. One reaction for one time point of amylase assay contained 20 μl of supernatant containing digestive enzymes or crude enzyme extract, 500 μl of 0.5% starch solution (solubilized in 0.05 M Tris-HCl, pH 8.0), and 480 μl of 0.2 M Tris-HCl, pH 8.0. For protease assay, one reaction contained 25 μl of crude enzyme extract, 250 μl of 1% casein (solubilized in 0.05 M Tris-HCl, pH 7.5), and 725 μl of 0.2 M Tris-HCl, pH 8.0. The reactions were incubated at room temperature (23 °C) for 5, 10, 15, 20, 25, or 30 min. Five hundred microliters of 3′, 5′-dinitrosalicylic acid (DNS) solution and 12% trichloroacetic acid were added to terminate amylase and protease reactions, respectively. The reaction mixtures were subsequently centrifuged at 16,000 × *g* for 10 min to sediment remaining substrate and denatured enzyme. Supernatants containing the catalytic products of amylase and protease were taken to measure absorbance at 540 and 280 nm, respectively. The absorbance values were plotted against the incubation time.

### Determination of optimum pH of enzyme reaction

The activities of amylase and protease were observed at pH 5–11 and pH 1–11, respectively. Buffers used (0.2 M) comprised KCl–HCl (pH 1.0), glycine–HCl (pH 2.0 and 3.0), sodium acetate (pH 4.0 and 5.0), sodium phosphate (pH 6.0 and 7.0), Tris-HCl (pH 8.0 and 9.0), and glycine–NaOH (pH 10.0 and 11.0). One milliliter of reaction for amylase assay contained 20 μl of crude enzyme extract, 500 μl of 0.5% starch solution (solubilized in 0.05 M Tris-HCl, pH 8.0), and 480 μl of 0.2 M buffer. After incubation at room temperature (23 °C) for 7 min, the reactions were stopped with 500 μl of DNS solution, boiled for 10 min, and centrifuged at 16,000 × *g* for 10 min. Absorbance of the supernatants was measured at 540 nm.

For protease assay, one reaction included 25 μl of crude enzyme extract, 250 μl of 1% casein (solubilized in 0.05 M Tris-HCl, pH 7.5), and 725 μl of 0.2 M buffer as described above. The reaction was allowed to proceed at room temperature (23 °C) for 7 min and was then terminated with 12% trichloroacetic acid. The reactions were incubated on ice for 30 min then centrifuged to separate denatured casein and enzyme. Absorbance of the resulting supernatants was measured at 280 nm.

### Determination of optimum temperature of enzyme reaction

Amylase and protease reactions were monitored at 29, 35, 40, 45, 50, 55, 60, and 70 °C. One milliliter of reaction for amylase assay contained 20 μl of crude enzyme extract, 500 μl of 0.5% starch solution (solubilized in 0.05 M Tris-HCl, pH 8.0), and 480 μl of 0.2 M of the optimum buffer (as determined in section “Determination of optimum enzyme reaction pH”). In assay of protease activity, the reaction contained 25 μl of crude enzyme extract, 250 μl of 1% casein (solubilized in 0.05 M Tris-HCl, pH 7.5), and 725 μl of optimum buffer. The reactions were incubated for 7 min at various temperatures (listed above). The reactions were terminated, incubated on ice for 30 min, and centrifuged at 16, 000 × *g* for 10 min. The supernatants were measured at 540 and 280 nm to determine the catalytic products of amylase and protease, respectively.

### Determination and comparison of amylase and protease activities in optimum and environmental conditions

Five to eight specimens of each fish species were determined for both amylase and protease activities. One milliliter of amylase reaction contained 20 μl of crude enzyme extract, 500 μl of 0.5% starch solution, and 480 μl of 0.2 M optimum buffer. The protease-assay reaction contained 25 μl of crude enzyme extract, 250 μl of 1% casein, and 725 μl of optimum buffer. The reactions were incubated for 10 min at the optimum temperature for each enzyme. DNS solution and 12% trichloroacetic acid were used to terminate the amylase and protease reactions, respectively. Reactions were incubated on ice for 30 min (for protease assay) or boiled for 10 min (for amylase assay) then centrifuged at 16,000 × *g* for 10 min to sediment remaining substrate and denatured enzyme. The resulting supernatants were taken to measure the absorbance at 540 and 280 nm, respectively. The contents of product released from substrate were calculated from tyrosine and maltose standard curves, respectively. The enzyme activity and specific activity were subsequently analyzed and compared between fish species. One unit of protease activity was defined as the amount of enzyme required to generate one nmol of tyrosine equivalents in 1 min; one unit of amylase activity was defined as the amount of enzyme required to produce one mmol of maltose in 1 min. Specific activity is the enzyme activity (nmol tyrosine equivalents min^-1^ for proteases or mmol maltose min^-1^ for amylase) per mg of total protein and therefore it was reported as units mg^-1^ protein.

The activities of both digestive enzymes were also determined at swamp temperature; the reaction components and procedure were similar to reactions in optimum conditions, but the assay temperature was changed to 29 °C for all samples.

Control reactions were also conducted for all samples. The reactions contained all components as described for the catalytic reaction, but the DNS solution or 12% trichloroacetic acid were pipetted into the reaction before adding crude enzyme. The obtained absorbance values were subtracted from those for catalytic reactions.

### Statistical analysis

Enzyme activities are expressed as mean ± SD. Data were analyzed by one-way analysis of variance (IBM SPSS statistics software v.19). Post-hoc multiple comparisons were performed using Duncan’s test. Differences were considered significant at *P* < 0.05.

### Determination of isoenzyme pattern and molecular weight analysis

Total crude enzymes (25 and 100 μg respectively) were used to analyze amylase and protease isoenzymes, by using sodium dodecyl sulfate (SDS)-polyacrylamide gel electrophoresis and zymography. Crude enzyme samples were mixed with SDS sample buffer without any reducing agent (62.5 mM Tris-HCl, pH 6.8, 25% glycerol, 2% SDS, 0.05% bromophenol blue) and loaded on a 5% stacking gel. The proteins were separated using a 12% separating gel. Electrophoresis was performed at 180 V and 4 °C using Mini-PROTEAN Tetra Cell apparatus (Bio-Rad).

After electrophoresis, two gels with a similar pattern of sample loading were separately examined. The first gel was stained with Coomassie Brilliant Blue [0.15% (w/v) Coomassie Brilliant Blue R-250, 50% ethanol, 10% glacial acetic acid] to observe the protein pattern. Staining was performed for 1–2 h and then destaining (30% ethanol, 10% glacial acetic acid) was carried out until the background was clear. The protein bands were dark blue. For in-gel analysis of amylase activity, the second gel was soaked for 20 min in 0.05 M Tris–HCl, pH 8.0, containing 2% Triton X-100 to remove SDS and washed for 5 min with the same buffer without Triton X-100. The gel was immersed in 2% soluble starch (solubilized in 0.05 M Tris-HCl, pH 8.0) and incubated at 4 °C for 30 min before moving to 45 °C. The enzyme was allowed to react in-gel for 1 h. The starch solution was discarded and the gel was rinsed with water. Iodine solution was used to stain the gel. The presence of starch produced a brownish–purple color on the gel. A clear band thus showed the position of amylase (i.e., degradation of the starch); such bands were used to determine enzyme molecular weight by comparison of retention factors with those of proteins of standard molecular weight on the Coomassie Brilliant Blue-stained gel.

Protein markers with different molecular weights were used in this study. M1 was Pink Plus-prestained protein ladder from GeneDirex and contained 11 proteins in the range 10–175 kDa. The manufacturer does not provide any detail about the specific proteins in this marker mixture. M2 was unstained protein molecular weight marker from Thermo Scientific, containing chicken egg white lysozyme (14.4 kDa), bovine milk β-lactoglobulin (18.4 kDa), *Escherichia coli* REase Bsp98I (25 kDa), porcine muscle lactate dehydrogenase (35 kDa), chicken egg white ovalbumin (45 kDa), bovine plasma bovine serum albumin (66.2 kDa), and *E. coli* β-galactosidase (116.0 kDa).

To detect protease activity in-gel, the procedure was similar to that described above for amylase. SDS was removed by using 0.05 M Tris–HCl buffer, pH 8.0, containing 2% Triton X-100. Gels were rinsed with the same buffer without Triton X-100 before soaking in 2% casein (solubilized in 0.05 M Tris–HCl, pH 8.0) at 4 °C for 30 min. Gels were then incubated with gentle shaking at 45 °C for 1 h. Casein solution was removed and the gels were rinsed with water before staining with Coomassie Brilliant Blue R-250. Protein staining was performed at room temperature for 1 to 2 h. Destaining was carried out for 30 min or until a clear band was visualized against the dark blue background. The molecular weight of proteases was analyzed as described above for amylases.

### Effects of incubation time, pH, and temperature on enzyme activity

Enzyme activity was determined after incubation of crude enzyme in 0.2 M Tris-HCl, pH 8.0, for 30, 60, 90, 120, or 150 min. At each time point, enzyme mixture was aliquoted and substrate solution was added (0.5% starch for amylase assay, or 1% casein for protease assay). The reaction was incubated at room temperature (23 °C). The enzymatic product was detected by measuring the absorbance as described above. The enzyme activity and relative activity were calculated; 100% relative activity was defined as the enzyme activity measured in the same conditions, without a pre-incubation step.

The effect of pH on enzyme activity was investigated. Crude enzyme was incubated for 1 h at room temperature (23 °C) in buffer at pH 5–11. Enzyme solution was aliquoted and mixed with the optimum buffer and substrate for activity testing. The residual activity of enzyme was monitored and is reported as % relative activity. The activities at the optimum pH without pre-incubation were used as control (100% of relative activity).

To determine the effect of temperature, crude enzyme mixed with 0.2 M Tris-HCl, pH 8.0, was incubated at 23–60 °C for 1 h. Substrate and the optimum buffer for reaction were mixed with the enzyme solution before assay at the optimum reaction temperature. The 100% of relative activity at optimum temperature, without pre-incubation step, was used as control.

## Supplementary Information

Supplementary Information
