# Supplementary material for: Diverse activities and biochemical properties of amylase and proteases from six freshwater fish species
Source: Sci Rep. 2021 Mar 11;11:5727. doi: 10.1038/s41598-021-85258-7 (PMC7970969; doi:10.1038/s41598-021-85258-7)
Supplement: Supplementary file 1 — Supplementary Information [file 41598_2021_85258_MOESM1_ESM.docx]

**Supplementary information**

Diverse activities and biochemical properties of amylase and proteases from six freshwater fish species

Chamaiporn Champasri^1^*, Suthathip Phetlum^1^, Chanakan Pornchoo^1^

**Figure 3.** All gels were the full-length gels and were the real and original ones from scanner without contrast adjustment. For the zymographic gels of amylase, the gel had to stain with iodine solution for 15 min and destained with distill water or tap water for 15-20 min. If prolong detaining, the amylase bands will turn to fade out. For alkaline proteases, the zymographic gels were stained with Coomassie brilliant blue R-250 for 1-2 h and destained for 30-40 min until the clear bands were observed against the dark blue background. Therefore they are the best zymographic gels to clearly observe the enzyme bands under our best optimization of staining and destaining time without any making contrast to the pictures.

Makers M1 was loaded onto the wells along with the samples for all Coomassie brilliant blue-stained gels. There are no available amylase and proteases markers for zymographic gels. Therefore we have to put the marker M1 and M2 on the left in order to estimate the size and the positions of the enzymes. Gels stained with Coomassie brilliant blue R-250 and substrate specific dye were set with the same length of separating gel and had the same migration distance or Rf of dye front.
